# First-line immune checkpoint inhibitors combined with targeted therapy for advanced renal cell carcinoma: a network meta-analysis and cost-effectiveness analysis

**DOI:** 10.3389/fimmu.2026.1912070

**Published:** 2026-09-30

**Authors:** Yize Jia, Shan Zhao, Xiaohui Wang, Shuo Kang

**Affiliations:** 1 School of Pharmacy, Hebei Medical University, Shijiazhuang, China; 2 Department of Oncology, The Second Hospital of Hebei Medical University, Shijiazhuang, China; 3 Pharmaceutical Management Research Center, Hebei Medical University, Shijiazhuang, China; 4 Medical Insurance Office, The Second Hospital of Hebei Medical University, Shijiazhuang, China

**Keywords:** cost-effectiveness analysis, immunotherapy, network meta-analysis, renal cell carcinoma, targeted therapy

## Abstract

**Objective:**

This study aimed to systematically evaluate the efficacy of first-line immune checkpoint inhibitors (ICIs) combined with targeted therapy for advanced renal cell carcinoma (RCC) and assess their cost-effectiveness from the perspective of the Chinese healthcare system.

**Methods:**

Phase III randomized controlled trials comparing ICI-targeted combinations versus sunitinib as first-line treatment for advanced RCC were identified through systematic searches of PubMed, EMBASE, and the Cochrane Library. A network meta-analysis (NMA) was performed to compare the hazard ratios (HRs) for progression-free survival (PFS) and overall survival (OS) among different regimens. The surface under the cumulative ranking curve (SUCRA) was calculated to rank treatment efficacy. Based on the NMA results, a partitioned survival model was developed for the cost-effectiveness analysis. Using the sunitinib arm from the CheckMate 9ER trial as the reference, parametric survival models were fitted to extrapolate survival outcomes over a 20-year time horizon. HRs from the NMA were applied to derive survival curves for other interventions. The model cycle length was 6 weeks. Costs included drug acquisition, follow-up, adverse event management, subsequent therapy, and palliative care. Utility values were sourced from published literature. An incremental cost-effectiveness ratio (ICER) was calculated against a willingness-to-pay (WTP) threshold of $40,332.35 per quality-adjusted life-year (QALY). Sensitivity and scenario analyses were conducted.

**Results:**

Seven trials involving 4,973 patients were included. The NMA showed that pembrolizumab plus lenvatinib ranked highest for PFS, whereas toripalimab plus axitinib ranked highest for OS. The cost-effectiveness analysis indicated that toripalimab plus axitinib yielded the greatest gains in life-years and QALYs, with the lowest ICER of $62,204.37 among all evaluated strategies. However, all regimens exceeded the WTP threshold of $40,332.35 per QALY. Sensitivity analyses confirmed the robustness of these findings.

**Conclusion:**

This study demonstrates that while toripalimab plus axitinib is the most efficacious first-line regimen for aRCC and offers the lowest ICER, none of the evaluated regimens are economically viable from the perspective of the Chinese healthcare system at the WTP threshold of $40,332.35 per QALY.

**Systematic review registration:**

https://www.crd.york.ac.uk/PROSPERO/view/CRD420261503119, identifier CRD420261503119.

## 1 Introduction

According to the 2022 GLOBOCAN report (1), kidney cancer ranks as the 14th most commonly diagnosed cancer and the 16th leading cause of cancer death worldwide. Pathologically, renal cell carcinoma (RCC) constitutes the predominant histological subtype of kidney cancer (2). In 2022, there were an estimated 73,700 new cases of renal cell carcinoma (RCC) in China. This number accounted for 1.5% of all new cancer cases. The estimated number of deaths caused by RCC was 24,000. This figure represented 0.9% of all cancer deaths in the country (3).

The prognosis of advanced renal cell carcinoma (aRCC) has improved markedly over the past decade due to advances in therapeutic modalities, yet it remains a challenging disease overall. For patients with aRCC, sunitinib was historically used as standard therapy (4). Although targeted agents—such as vascular endothelial growth factor (VEGF), mammalian target of rapamycin (mTOR) inhibitors, and tyrosine kinase inhibitors (TKIs)—have constituted the first-line standard for aRCC for the past decade, the current treatment paradigm has shifted toward immune-based combinations. In recent years, targeted-immunotherapy combinations have become the first-line standard for aRCC (5, 6). For instance, the combination of axitinib and toripalimab extended the median progression-free survival (mPFS) from 9.8 months to 18.0 months (7) and reduced the risk of death by 39%. Additionally, the combination of nivolumab and cabozantinib achieved a median overall survival (mOS) of 45.3 months (8). However, head-to-head randomized controlled trials directly comparing all these regimens are lacking. Therefore, a network meta-analysis (NMA) is required to perform indirect comparisons of their relative efficacy in the absence of direct evidence, thereby providing evidence for clinical decision-making.

Although these novel immune-targeted combination therapies demonstrate satisfactory efficacy, they incur extremely high costs and impose a substantial economic burden on patients and healthcare systems. In the context of limited medical resources, especially in upper-middle-income countries such as China, evidence of clinical efficacy alone is insufficient to justify widespread clinical application. Therefore, it is necessary to evaluate the cost-effectiveness of these regimens from a health economics perspective. This assessment can provide critical supplementary evidence for medical insurance policymaking, hospital management, and individualized treatment decisions by clinicians.

In summary, this study aims to systematically compare the efficacy of different first-line immunotherapy plus targeted therapy combinations for aRCC through NMA. On this basis, a cost-effectiveness analysis of the optimal treatment regimens will be conducted from the perspective of China’s healthcare system. The findings intend to offer comprehensive evidence for clinical practice and the rational allocation of health resources.

## 2 Methods

### 2.1 Network meta-analysis

#### 2.1.1 Search strategy

We conducted the NMA in accordance with the PRISMA guidelines (**Supplementary Table S1**) (9). We systematically searched PubMed, EMBASE, and the Cochrane Library to identify phase III randomized controlled trials (RCTs) evaluating first-line combinations of immune checkpoint inhibitors (ICIs) with targeted agents for patients with aRCC. The search was conducted from inception until March 1, 2026. We also manually searched conference abstracts from major international oncology meetings, including the American Society of Clinical Oncology (ASCO), the European Society for Medical Oncology (ESMO), and the American Association for Cancer Research (AACR) (**Supplementary Methods**).

#### 2.1.2 Study screening and data extraction

The study selection process was conducted independently by two investigators to minimize bias. Initially, all records retrieved from the database searches were imported into reference management software (EndNote X9), and duplicates were removed automatically and manually. Subsequently, the titles and abstracts of the remaining records were screened against the pre-defined eligibility criteria (**Supplementary Table S2**). The full texts of all potentially relevant articles were then obtained and assessed in detail. Any disagreements between the two reviewers at any stage of the screening process were resolved through discussion or by consultation with a third senior investigator if necessary. The entire screening process was documented and presented in a PRISMA flow diagram (**Supplementary Figure S1**).

Data from the included studies were extracted independently by the same two investigators using a standardized, pilot-tested data extraction form developed in Microsoft Excel. The extracted information included: first author, publication year, clinical trial registration number, study phase, design, sample size, drugs, hazard ratios (HRs) with 95% confidence intervals (CIs) for progression-free survival (PFS) and overall survival (OS) and incidence of grade ≥3 adverse events.

#### 2.1.3 Risk of bias assessment

The risk of bias in the included randomized controlled trials was assessed independently by two investigators using the revised Cochrane Risk of Bias tool for randomized trials (RoB 2.0).

#### 2.1.4 Synthesis of data and statistical analysis

The relative efficacy of all first-line treatment regimens was evaluated using a network meta-analysis. All NMA procedures were performed using R software (version 4.4.2) with the gemtc package. A fixed-effect model was used because each direct treatment comparison was based on a single phase III randomized controlled trial and between-study heterogeneity could not be estimated. Therefore, I² and τ² were not available. The evidence network was star-shaped, with sunitinib as the common comparator, and no closed loops were present. Accordingly, node-splitting and other loop-based consistency assessments were not applicable (10, 11). The network structure is shown in **Figure 1**. The hierarchy of treatments was evaluated by calculating the surface under the cumulative ranking (SUCRA) curve for each intervention, representing the probability of it being the most effective option.

**Figure 1:**
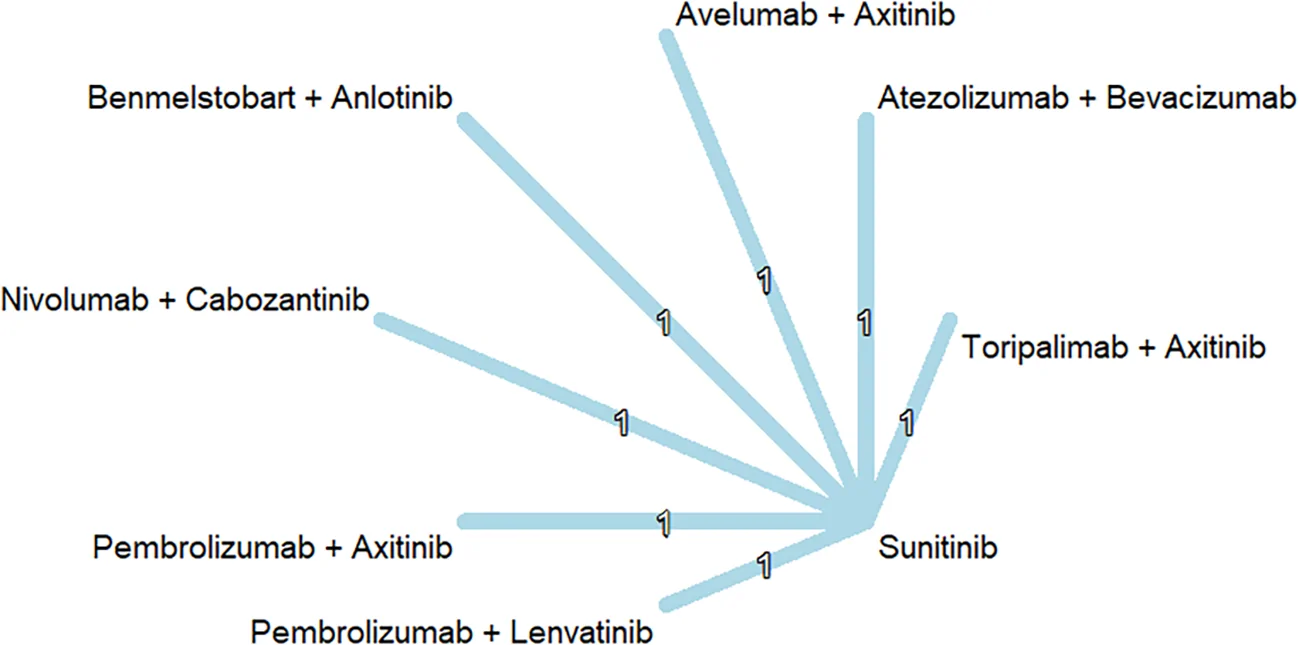
Network plot of the network meta-analysis.

### 2.2 Cost-effectiveness analysis

#### 2.2.1 Model structure

This cost-effectiveness analysis followed the Consolidated Health Economic Evaluation Reporting Standards (CHEERS) checklist (**Supplementary Table S3**) (12). A three-state partitioned survival model (**Figure 2**) was developed to estimate the long-term clinical and economic outcomes of the evaluated first-line strategies. The model comprised PFS, progressed disease (PD), and death. At model entry, all patients were assumed to be in the PFS state. The model used a 6-week cycle, consistent with the treatment administration schedule. The analysis was conducted over a 20-year time horizon to capture long-term outcomes because this time horizon can capture the majority of patients’ survival events. An annual discount rate of 5% was applied to both future costs and health outcomes (13). The impact of the discount rate was further examined in the sensitivity analysis.

**Figure 2:**
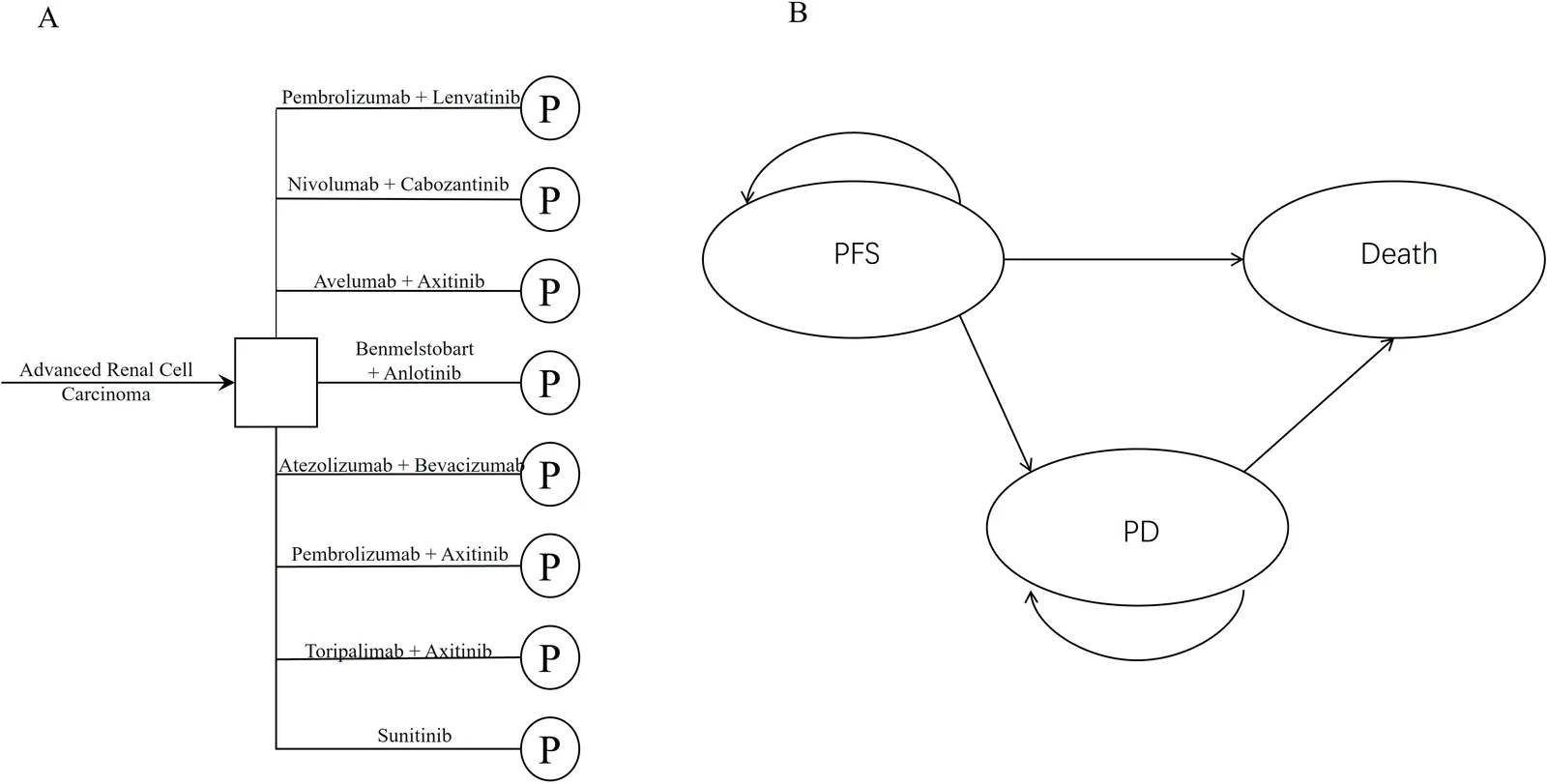
The structure of the **(A)** decision tree and **(B)** partitioned survival model. PFS, progression-free survival; PD, progressed disease.

#### 2.2.2 Clinical data inputs

Clinical inputs were obtained from the included trials and the NMA. The sunitinib arm of CheckMate 9ER was used as the reference survival source because this trial provided relatively mature follow-up data (14). The uncertainty associated with the selection of CheckMate 9ER as the baseline survival source was further examined in scenario analyses using the sunitinib arms from alternative phase III trials. Published K-M curves for the sunitinib arm of CheckMate 9ER were digitized using GetData Graph Digitizer (v2.26) to obtain survival coordinates. Pseudo-individual patient data were then reconstructed in R (v4.4.2) and fitted using a range of candidate survival models, including exponential, Weibull, gamma, generalized gamma, Gompertz, log-logistic, log-normal, and Royston-Parmar (RP) spline models (15, 16). The optimal parametric distribution was determined based on the Akaike information criterion (AIC) and Bayesian information criterion (BIC), with lower values indicating a better fit (17). Model fit was further assessed by visual comparison of the fitted and observed survival curves (**Supplementary Figures S2**–**S15**). AIC values and BIC values were shown in **Supplementary Table S4** and the survival parameters for selected model were shown in **Supplementary Table S5**. For the remaining ICI-targeted regimens, long-term survival was estimated by applying the NMA-derived HRs as exponents to the fitted sunitinib survival curve. After disease progression, everolimus was assumed as the subsequent treatment, with the proportion receiving second-line therapy obtained from the corresponding clinical trials (18). The impact of this assumption was examined in scenario analyses using alternative subsequent-treatment regimens.

#### 2.2.3 Cost and utility

Only direct medical costs were considered from the perspective of the Chinese healthcare system. These included drug acquisition costs, supportive treatment, routine follow-up, subsequent therapy, terminal care cost, and management of treatment-related adverse events (AEs). A patient body weight of 65kg was assumed for calculating drugs dosing (19). The utility values for the health states of PFS, PD, and death were set at 0.78, 0.66, and 0 respectively (19). All input parameters and distributions are detailed in **Table 1** (7, 8, 13, 19, 21–31).

**Table 1:** Model input parameters and ranges used in the sensitivity analysis.

Parameter	Base-line value	Range		Reference
		Lower bound	Upper bound	
Cost values (US $)				
Sunitinib per 12.5mg	2.53	1.90	3.16	Local charge
Atezolizumab per 1200mg	4605.45	3454.09	5756.81	Local charge
Bevacizumab per 100mg	139.29	104.47	174.11	Local charge
Avelumab per 10mg	81.74	61.31	102.18	(22)
Axitinib per 1 mg	8.05	6.04	10.07	Local charge
Benmelstobart per 600 mg	1724.23	1293.18	2155.29	Local charge
Anlotinib per 12mg	39.81	29.85	49.76	Local charge
Nivolumab per 40mg	644.02	483.02	805.03	Local charge
Cabozantinib per 60mg	491.30	368.47	614.12	(22)
Ipilimumab per 50mg	2527.38	1895.53	3159.22	Local charge
Pembrolizumab per 100mg	2515.87	1886.90	3144.83	Local charge
Lenvatinib per 4mg	1.19	0.89	1.49	Local charge
Toripalimab per 240mg	264.65	198.49	330.82	Local charge
Everolimus per 2.5mg	6.78	5.09	8.48	Local charge
Routine follow-up per cycle	442.30	331.725	552.875	(23)
Best supportive care per cycle	92.23	69.1725	115.2875	(24)
Terminal care per event	1893.00	1419.75	2366.25	(19)
Increased lipase per event	0	0	0.00	(25)
Hypertension per event	12.61	9.46	15.76	(26)
Diarrhea per event	43.3	32.48	54.13	(26)
Platelet count decreased per event	254.02	190.52	317.53	(27)
White blood cell decreased per event	89	66.92	111.54	(27)
ALT increased per event	25.09	18.82	31.36	(26)
Anemia per event	508.2	381.15	635.25	(19)
Proteinuria per event	121.65	91.24	152.06	(26)
Utility values				
PFS	0.78	0.585	0.975	(19)
PD	0.66	0.495	0.825	(19)
Probability of SAEs in Sunitinib group				
Hypertension	0.212	0.159	0.265	(8)
Platelet count decreased	0.239	0.179	0.298	(8)
White blood cell decreased	0.117	0.088	0.147	(8)
Probability of SAEs in Pembrolizumab plus Lenvatinib group				
Diarrhea	0.111	0.083	0.139	(28)
Hypertension	0.290	0.217	0.362	(28)
Probability of SAEs in Nivolumab plus Cabozantinib group				
Hypertension	0.131	0.098	0.164	(8)
Probability of SAEs in Avelumab + Axitinib group				
Hypertension	0.277	0.207	0.346	(29)
Probability of SAEs in Benmelstobart plus Anlotinib group				
Hypertension	0.345	0.259	0.431	(30)
Platelet count decreased	0.008	0.006	0.010	(30)
White blood cell decreased	0.008	0.006	0.010	(30)
Probability of SAEs in Atezolizumab plus Bevacizumab group				
Hypertension	0.140	0.105	0.175	(31)
Probability of SAEs in Pembrolizumab plus Axitinib group				
Diarrhoea	0.107	0.080	0.134	(32)
Hypertension	0.221	0.166	0.277	(32)
ALT increased	0.126	0.094	0.157	(32)
Probability of SAEs in Toripalimab plus Axitinib group				
Neutropenia	0.111	0.083	0.138	(7)
Anemia	0.154	0.115	0.192	(7)
Thrombocytopenia	0.289	0.216	0.361	(7)
Leukopenia	0.010	0.007	0.012	(7)
Neutrophil count decreased	0.000	0.000	0.000	(7)
Proportion of patients received second-line therapy				
Sunitinib group	0.554	0.416	0.693	(8)
Pembrolizumab + Lenvatinib group	0.510	0.382	0.637	(28)
Nivolumab + Cabozantinib group	0.431	0.324	0.539	(8)
Avelumab + Axitinib group	0.581	0.436	0.727	(29)
Benmelstobart + Anlotinib group	0.258	0.193	0.322	(30)
Atezolizumab + Bevacizumab group	0.425	0.319	0.531	(31)
Pembrolizumab + Axitinib group	0.549	0.411	0.686	(32)
Toripalimab + Axitinib group	0.162	0.121	0.202	(7)
Other				
Discount rate	5%	0%	8%	(13)
Body weight	65.00	48.75	81.25	(19)

#### 2.2.4 Base case analysis

Effectiveness was expressed in quality-adjusted life years (QALYs). All costs were converted to 2024 U.S. dollars using an exchange rate of US $1 = CNY 7.122, and both costs and health outcomes were discounted annually at 5%. Cost-effectiveness was evaluated using the ICER, with a WTP threshold of $40,332.35 per QALY based on the World Health Organization (WHO) recommendations (20) and China guidelines for pharmacoeconomic evaluations (13). Cost and utility parameters were shown in **Table 1**.

#### 2.2.5 Sensitivity analysis

Uncertainty in the model outcomes was explored through one-way and probabilistic sensitivity analyses. In the one-way analysis, individual parameters were varied by ±25% from their base-case values, and the resulting changes in the ICER were summarized using a tornado diagram. For the probabilistic analysis, 1,000 Monte Carlo simulations were performed to generate cost-effectiveness acceptability curves. Cost-effectiveness probabilities were evaluated at WTP thresholds of $13,444.12, $26,888.23, and $40,332.35 per QALY, corresponding to 1, 2, and 3 times China’s 2024 per capita GDP, respectively. For this analysis, cost data were assigned a gamma distribution, while utility values and adverse event incidence rates were assigned beta distributions.

#### 2.2.6 Scenario analysis

Several scenario analyses were conducted to assess uncertainty related to key model assumptions. Alternative baseline survival scenarios were examined using the sunitinib arms from CLEAR, JAVELIN Renal 101, IMmotion151, KEYNOTE-426, ETER100, and RENOTORCH instead of CheckMate 9ER. For each alternative baseline, PFS and OS curves were reconstructed from the published Kaplan–Meier curves and fitted independently using the candidate survival models described above. Model selection was primarily based on the lowest AIC, supplemented by visual inspection of the fitted curves and the clinical plausibility of long-term extrapolation (**Supplementary Figures S2**–**S15**). All other model inputs were kept unchanged.

Alternative subsequent-treatment scenarios were evaluated to assess the impact of the base-case assumption of everolimus after disease progression. Pazopanib, sorafenib, and vorolanib plus everolimus were considered as alternative subsequent treatments. Only subsequent-treatment costs were changed in these scenarios, while survival outcomes and other model inputs remained unchanged.

To explore the potential effect of future drug-price negotiations in China, price reductions of 20%, 40%, and 60% were applied to drugs not included in the 2025 National Reimbursement Drug List (NRDL) (21). For regimens containing two non-NRDL drugs, the prices of both agents were reduced by the same proportion, whereas the prices of NRDL-listed drugs were kept unchanged.

A price-threshold analysis was performed to estimate the minimum price reduction required for each combination regimen to become cost-effective. In this analysis, the prices of both drugs within each combination regimen were assumed to decrease simultaneously by the same proportion, irrespective of their current NRDL status, until the ICER reached the prespecified WTP threshold of $40,332.35 per QALY.

## 3 Results

### 3.1 Network meta-analysis results

A total of 14,688 records were initially identified through database searches. After the selection process (**Supplementary Figure S1**), 7 trials (7, 8, 28–32) (CLEAR, CheckMate 9ER, JAVELIN Renal 101, ETER-100, IMmotion151, KEYNOTE-426, RENOTORCH) were included in the NMA. **Supplementary Table S6** summarizes the characteristics of these trials. The overall risk of bias for the included studies is summarized in **Supplementary Figure S16**. The HRs for each treatment regimen are presented in **Table 2**. For PFS, pembrolizumab plus lenvatinib showed the highest SUCRA ranking, whereas toripalimab plus axitinib ranked highest for OS (**Figures 3A, B**).

**Table 2:** Hazard ratios of the network meta-analysis of the progression-free survival and overall survival.

Overall survival							
Treatment	Ate+Bev	Ava+Axi	Ben+Anl	Nivo+Cabo	Pem+Axi	Pem+Len	Tori+Axi
Ate+Bev	1	0.97 (0.76–1.23)	0.72 (0.50–1.05)	0.87 (0.67–1.12)	0.93 (0.72–1.18)	0.87 (0.65–1.15)	0.67 (0.43–1.05)
Ava+Axi	1.03 (0.81–1.31)	1	0.75 (0.52–1.08)	0.90 (0.69–1.16)	0.95 (0.76–1.20)	0.90 (0.68–1.19)	0.69 (0.44–1.09)
Ben+Anl	1.38 (0.95–2.00)	1.33 (0.93–1.92)	1	1.19 (0.82–1.75)	1.27 (0.88–1.82)	1.19 (0.81–1.79)	0.93 (0.54–1.56)
Nivo+Cabo	1.15 (0.89–1.50)	1.11 (0.86–1.44)	0.84 (0.57–1.22)	1	1.06 (0.82–1.37)	1.00 (0.74–1.35)	0.77 (0.49–1.22)
Pem+Axi	1.08 (0.85–1.38)	1.05 (0.83–1.32)	0.79 (0.55–1.13)	0.94 (0.73–1.22)	1	0.94 (0.71–1.25)	0.72 (0.46–1.14)
Pem+Len	1.15 (0.87–1.53)	1.11 (0.84–1.47)	0.84 (0.56–1.24)	1.00 (0.74–1.35)	1.06 (0.80–1.41)	1	0.77 (0.48–1.23)
Tori+Axi	1.49 (0.95–2.34)	1.44 (0.92–2.26)	1.08 (0.64–1.84)	1.30 (0.82–2.05)	1.38 (0.88–2.16)	1.30 (0.81–2.08)	1
Progression-Free survival							
Treatment	Ate+Bev	Ava+Axi	Ben+Anl	Nivo+Cabo	Pem+Axi	Pem+Len	Tori+Axi
Ate+Bev	1	0.79 (0.64–0.99)	0.64 (0.48–0.85)	0.70 (0.55–0.89)	0.83 (0.66–1.04)	0.56 (0.44–0.74)	0.78 (0.56–1.09)
Ava+Axi	1.26 (1.01–1.57)	1	0.80 (0.61–1.06)	0.88 (0.69–1.11)	1.04 (0.84–1.30)	0.71 (0.55–0.92)	0.98 (0.71–1.35)
Ben+Anl	1.57 (1.18–2.08)	1.25 (0.94–1.65)	1	1.10 (0.81–1.47)	1.30 (0.98–1.72)	0.88 (0.65–1.20)	1.22 (0.85–1.75)
Nivo+Cabo	1.43 (1.12–1.82)	1.14 (0.90–1.44)	0.91 (0.68–1.23)	1	1.19 (0.93–1.52)	0.82 (0.62–1.06)	1.12 (0.81–1.56)
Pem+Axi	1.20 (0.96–1.51)	0.96 (0.77–1.19)	0.77 (0.58–1.02)	0.84 (0.66–1.07)	1	0.68 (0.53–0.88)	0.94 (0.68–1.30)
Pem+Len	1.77 (1.36–2.29)	1.40 (1.09–1.81)	1.13 (0.83–1.54)	1.22 (0.94–1.62)	1.47 (1.14–1.90)	1	1.39 (0.98–1.96)
Tori+Axi	1.28 (0.92–1.77)	1.02 (0.74–1.40)	0.82 (0.57–1.18)	0.89 (0.64–1.24)	1.06 (0.77–1.47)	0.72 (0.51–1.02)	1

Ate, Atezolizumab; Bev, Bevacizumab; Ava, Avelumab; Axi, Axitinib; Ben, Benmelstobart; Anl, Anlotinib; Nivo, Nivolumab; Cabo, Cabozantinib; Ipi, Ipilimumab; Pem, Pembrolizumab; Len, Lenvatinib; Sun, Sunitinib; Tori, Toripalimab.

**Figure 3:**
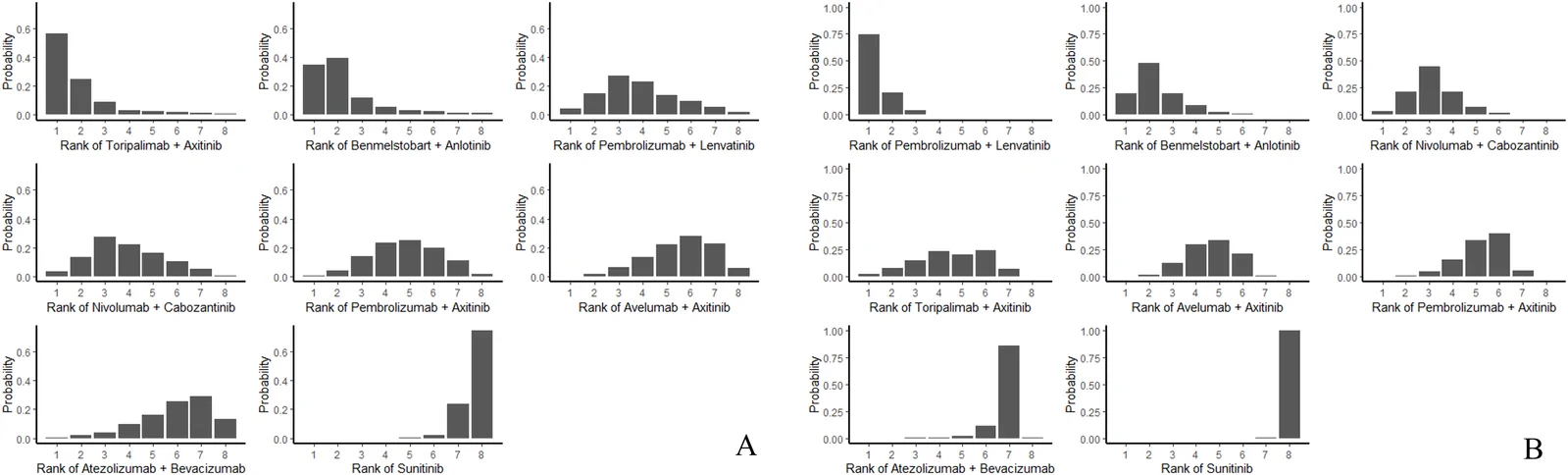
Ranking probabilities of different treatment regimens for **(A)** overall survival and **(B)** progression-free survival. OS, overall survival; PFS, progression-free survival.

### 3.2 Cost-effectiveness analysis results

#### 3.2.1 Base-case analysis results

From the perspective of the Chinese healthcare system and over a 20-year timeframe, the combination of toripalimab and axitinib provided the greatest gain in QALYs. Base-case analysis revealed that none of the evaluated first-line regimens were deemed cost-effective at the Chinese WTP threshold of $40,332.35 per QALY. Among all ICI plus targeted therapy regimens, toripalimab plus axitinib demonstrated the lowest ICER at $62,204.37 per QALY gained, yet it still exceeded the predefined threshold. Detailed results of the base-case analysis are presented in **Table 3**.

**Table 3:** Base-case analysis results.

Treatment	Costs ($)	QALYs	Incremental cost ($)	Incremental QALYs	ICER ($/QALY)
Sunitinib	108691.1057	3.9749	NA	NA	NA
Toripalimab + Axitinib	214070.9886	5.6690	105379.8829	1.6941	62204.3704
Atezolizumab + Bevacizumab	310112.0995	4.3187	201420.9938	0.3438	585810.0613
Benmelstobart + Anlotinib	354575.3268	5.5053	245884.2212	1.5304	160662.8444
Pembrolizumab + Axitinib	409960.3006	4.6320	301269.1949	0.6571	458461.2052
Pembrolizumab + Lenvatinib	417349.3324	4.9891	308658.2267	1.0142	304325.3285
Avelumab + Axitinib	533928.9403	4.5006	425237.8346	0.5257	808897.9354
Nivolumab + Cabozantinib	952227.5316	4.8945	843536.4259	0.9196	917315.6286

LYs, Life-years; QALYs, Quality-adjusted life-years; ICER, Incremental cost-effectiveness ratio.

#### 3.2.2 Sensitivity analysis results

Drawing on the findings of the base-case analysis, one-way sensitivity analyses were conducted on key parameters for toripalimab plus axitinib versus sunitinib (**Figure 4**). The one-way sensitivity analysis showed that the discount rate was the most influential parameter, followed by the cost of axitinib and the utility values of the PFS and PD states. These parameters produced the largest variations in the ICER, whereas the remaining parameters had relatively limited effects. Probabilistic sensitivity analyses showed consistently low probabilities of cost-effectiveness across WTP thresholds corresponding to 1, 2, and 3 times China’s per capita GDP (**Figure 5**). Even at the highest threshold of $40,332.35 per QALY (3 times per capita GDP), no regimen achieved a cost-effectiveness probability exceeding 2%. Toripalimab plus axitinib had the most favorable economic performance among the evaluated regimens, with a base-case ICER of $62,204.37 per QALY and a cost-effectiveness probability of approximately 2% at this threshold. This confirms that, despite offering the greatest health benefits, none of the regimens represented an economically viable option for the Chinese healthcare system at current pricing levels.

**Figure 4:**
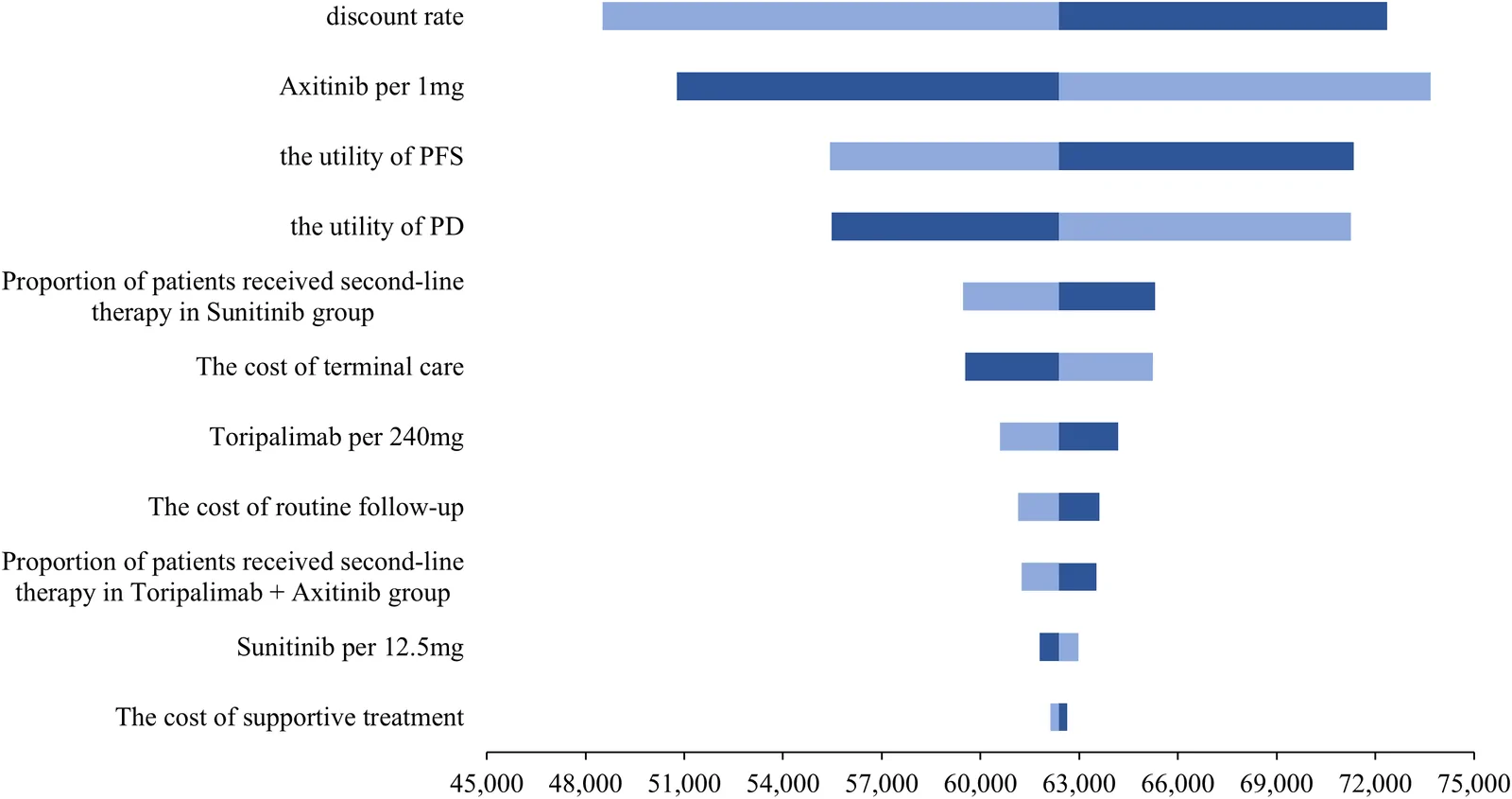
Tornado diagram of one-way sensitivity analysis for toripalimab plus axitinib versus sunitinib. PFS, progression-free survival; PD, progressed disease.

**Figure 5:**
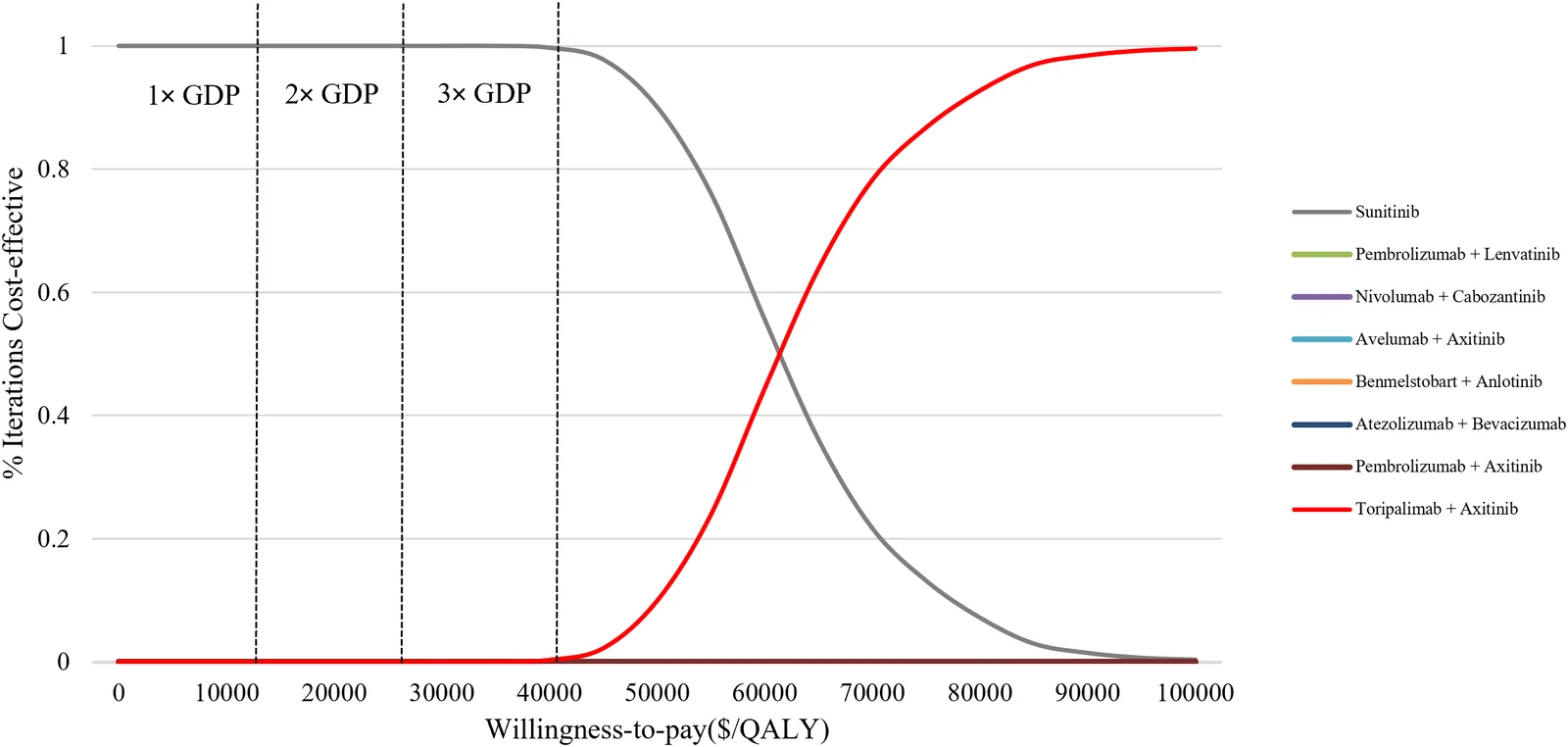
Cost-effectiveness acceptability curves of immune checkpoint inhibitors combined with targeted therapy. GDP, gross domestic product; QALY, quality-adjusted life-year.

#### 3.2.3 Scenario analysis results

The alternative baseline survival scenarios resulted in some variation in estimated costs, QALYs, and ICERs, but did not change the overall cost-effectiveness conclusions. For toripalimab plus axitinib, the ICER ranged from approximately $65,595 to $108,640 per QALY across the alternative baseline survival scenarios, compared with $62,204.37 per QALY in the base case. The largest deviation was observed when the RENOTORCH sunitinib arm was used as the baseline survival source. None of the evaluated regimens was cost-effective at the WTP threshold of $40,332.35 per QALY under any alternative baseline assumption (**Supplementary Table S7**).

Changes in subsequent-treatment assumptions affected total costs and ICERs, while QALYs remained unchanged. For toripalimab plus axitinib, the ICER was $57,026.85 per QALY under the pazopanib scenario, $68,132.11 per QALY under the sorafenib scenario, and $56,900.05 per QALY under the vorolanib plus everolimus scenario. The overall cost-effectiveness conclusions remained unchanged across the alternative subsequent-treatment scenarios (**Table 4**).

**Table 4:** Results of NRDL-related price-reduction scenario analysis.

Treatment regimen	Drug(s) subject to price reduction	Base-case ICER ($/QALY)	20% price reduction	40% price reduction	60% price reduction
Pembrolizumab + Lenvatinib	Pembrolizumab	304325.3285	240336.8842	176348.4398	112359.9955
Nivolumab + Cabozantinib	Nivolumab + Cabozantinib	917315.6286	849640.6730	545570.3541	359697.7169
Avelumab + Axitinib	Avelumab	808897.9354	672187.1952	535476.4549	398765.7147
Benmelstobart + Anlotinib	Benmelstobart	160662.8444	134954.612	109246.3796	83538.14716
Atezolizumab + Bevacizumab	Atezolizumab	585810.0613	492167.9312	398525.8011	304883.6709
Pembrolizumab + Axitinib	Pembrolizumab	458461.2052	392708.3469	326955.4886	261202.6304

Reducing the prices of non-NRDL drugs by 20%, 40%, and 60% progressively lowered the ICERs of the corresponding regimens. However, none of the evaluated regimens became cost-effective even when the prices of non-NRDL drugs were reduced by 60% (**Table 5**).

**Table 5:** Results of price-threshold analysis.

Treatment regimen	Drugs subject to simultaneous price reduction	Required simultaneous price reduction to reach WTP
Pembrolizumab + Lenvatinib	Pembrolizumab + Lenvatinib	92.8%
Nivolumab + Cabozantinib	Nivolumab + Cabozantinib	98.7%
Avelumab + Axitinib	Avelumab + Axitinib	97.6%
Benmelstobart + Anlotinib	Benmelstobart + Anlotinib	80.6%
Atezolizumab + Bevacizumab	Atezolizumab + Bevacizumab	90.0%
Pembrolizumab + Axitinib	Pembrolizumab + Axitinib	95.2%
Toripalimab + Axitinib	Toripalimab + Axitinib	41.5%

The price-threshold analysis showed that the simultaneous price reductions required for the combination regimens to reach the WTP threshold ranged from 41.5% to 98.7% (**Table 6**). Toripalimab plus axitinib required the smallest price reduction, at 41.5%, whereas nivolumab plus cabozantinib required the largest reduction, at 98.7%. All other regimens required price reductions of more than 80% to achieve cost-effectiveness.

**Table 6:** Scenario analyses under alternative second-line treatment assumptions.

Treatment regimen	Total cost (US$)	Incremental cost (US$)	QALYs	Incremental QALYs	ICER (US$/QALY)
Second-line scenario: Pazopanib					
Sunitinib	123,009.48	NA	3.9749	NA	NA
Toripalimab + Axitinib	219,618.17	96,608.68	5.6690	1.6941	57,026.85
Atezolizumab + Bevacizumab	321,202.63	198,193.14	4.3187	0.3438	576,422.23
Benmelstobart + Anlotinib	361,577.76	238,568.27	5.5053	1.5304	155,882.54
Pembrolizumab + Axitinib	423,858.30	300,848.81	4.6320	0.6571	457,821.48
Pembrolizumab + Lenvatinib	426,737.61	303,728.12	4.9891	1.0142	299,464.43
Avelumab + Axitinib	547,286.39	424,276.91	4.5006	0.5257	807,070.04
Nivolumab + Cabozantinib	962,411.73	839,402.25	4.8945	0.9196	912,819.86
Second-line scenario: Sorafenib					
Sunitinib	92,298.02	NA	3.9749	NA	NA
Toripalimab + Axitinib	207,720.03	115,422.01	5.6690	1.6941	68,132.11
Atezolizumab + Bevacizumab	297,414.58	205,116.55	4.3187	0.3438	596,558.17
Benmelstobart + Anlotinib	346,558.26	254,260.23	5.5053	1.5304	166,135.80
Pembrolizumab + Axitinib	394,048.51	301,750.49	4.6320	0.6571	459,193.62
Pembrolizumab + Lenvatinib	406,600.71	314,302.69	4.9891	1.0142	309,890.56
Avelumab + Axitinib	518,636.02	426,338.00	4.5006	0.5257	810,990.69
Nivolumab + Cabozantinib	940,567.66	848,269.64	4.8945	0.9196	922,462.83
Second-line scenario: Vorolanib + Everolimus					
Sunitinib	123,360.14	NA	3.9749	NA	NA
Toripalimab + Axitinib	219,754.02	96,393.88	5.6690	1.6941	56,900.05
Atezolizumab + Bevacizumab	321,474.23	198,114.09	4.3187	0.3438	576,192.32
Benmelstobart + Anlotinib	361,749.25	238,389.11	5.5053	1.5304	155,765.47
Pembrolizumab + Axitinib	424,198.66	300,838.52	4.6320	0.6571	457,805.81
Pembrolizumab + Lenvatinib	426,967.53	303,607.39	4.9891	1.0142	299,345.39
Avelumab + Axitinib	547,613.51	424,253.38	4.5006	0.5257	807,025.28
Nivolumab + Cabozantinib	962,661.14	839,301.00	4.8945	0.9196	912,709.76

QALY, quality-adjusted life-year; ICER, incremental cost-effectiveness ratio; NA, not applicable.

## 4 Discussion

This study provides an updated integrated assessment of the comparative efficacy and cost-effectiveness of first-line ICI plus targeted therapy regimens for aRCC. Our NMA showed that pembrolizumab plus lenvatinib achieved the highest SUCRA ranking for PFS, whereas toripalimab plus axitinib ranked highest for OS. These findings suggest that treatment selection in clinical practice should consider different therapeutic objectives. Pembrolizumab plus lenvatinib may be preferred when delaying disease progression is a major treatment goal, whereas toripalimab plus axitinib may represent a promising option when long-term survival benefit is prioritized. Nevertheless, these rankings are mainly based on indirect comparisons and should be interpreted together with safety, patient characteristics, treatment accessibility, and other clinical considerations.

Our findings extend those of previous network meta-analyses of first-line treatment for aRCC. Su et al. (11) included four randomized controlled trials and reported that pembrolizumab plus axitinib ranked favorably among the available ICI-based regimens in the overall population. In contrast, the present study incorporated seven phase III trials and included several more recently available ICI-targeted combinations, thereby providing an updated comparison of the current treatment landscape. In addition, whereas the previous economic evaluation was conducted from the US payer perspective, our analysis was performed from the perspective of the Chinese healthcare system, making the findings more directly relevant to clinical and reimbursement decision-making in China.

However, our cost-effectiveness analysis revealed a striking conflict between clinical efficacy and economic viability. Although toripalimab plus axitinib achieved the greatest gains in QALYs, its high drug cost resulted in an ICER exceeding the Chinese WTP threshold. These results are highly consistent with previous pharmacoeconomic studies on first-line immunotherapy combinations for aRCC (26), which consistently concluded that none of the current first-line immune-targeted regimens are cost-effective under current pricing. Even though toripalimab plus axitinib showed the most favorable economic profile among all included strategies, its cost-effectiveness probability remained extremely low. Collectively, these findings reinforce that high treatment costs represent a major barrier to the widespread clinical adoption of these effective regimens, and clinical benefit alone cannot justify their extensive application in the Chinese healthcare setting.

This study has several limitations. First, the network meta-analysis was based on published aggregate data, and a fixed-effect model was employed due to network structure constraints, which may not have fully captured between-study heterogeneity. Second, cost data were primarily based on publicly listed prices and medical service fees in mainland China, which may not be generalizable to other regions or payment systems. Third, the model assumed that all patients received the same second-line therapy after disease progression, without considering the potential impact of subsequent treatment lines on long-term survival and economic outcomes. To further explore this uncertainty, scenario analyses using alternative subsequent-treatment assumptions were conducted, and the overall cost-effectiveness conclusions remained unchanged. Finally, the economic evaluation was not conducted for patient subgroups, among whom efficacy and resource consumption may differ.

## 5 Conclusion

Integrating findings from both the NMA and the cost-effectiveness analysis, this study concludes that for the first-line treatment of aRCC in China, toripalimab plus axitinib represents the most efficacious therapeutic strategy based on current evidence. However, from the perspective of the Chinese healthcare system, none of the evaluated regimens were deemed cost-effective at the WTP threshold of $40,332.35 per QALY gained. Although toripalimab plus axitinib demonstrated the lowest ICER and the highest cost-effectiveness probability among all strategies, its economic viability remains unestablished. Consequently, clinical decision-makers face a dilemma rather than a trade-off: while toripalimab plus axitinib offers the greatest clinical benefit, its current pricing imposes a substantial financial burden. It is suggested that price negotiations or patient assistance programs are urgently needed to bridge the gap between clinical efficacy and economic feasibility. Future real-world studies are warranted to further validate the long-term effectiveness and economic impact of these regimens.

## Data Availability

The original contributions presented in the study are included in the article/**Supplementary Material**. Further inquiries can be directed to the corresponding author.
