# Supplementary material for: First-line immune checkpoint inhibitors combined with targeted therapy for advanced renal cell carcinoma: a network meta-analysis and cost-effectiveness analysis
Source: Front Immunol. 2026 Sep 30;17:1912070. doi: 10.3389/fimmu.2026.1912070 (PMC13661560; doi:10.3389/fimmu.2026.1912070)
Supplement: Supplementary file 1 [file DataSheet1.docx]

Supplementary Table S1 PRISMA Checklist

| **Section/Topic** | **Item #** | **Checklist Item** | **Reported on Page #** |
| --- | --- | --- | --- |
| **TITLE** |  |  |  |
| Title | 1 | Identify the report as a systematic review *incorporating a network meta-analysis (or related form of meta-analysis).* | **Page 1** |
|  |  |  |  |
| **ABSTRACT** |  |  |  |
| Structured summary | 2 | Provide a structured summary including, as applicable:  **Background:** main objectives  **Methods:** data sources; study eligibility criteria, participants, and interventions; study appraisal; and *synthesis methods, such as network meta-analysis.*  **Results:** number of studies and participants identified; summary estimates with corresponding confidence/credible intervals; *treatment rankings may also be discussed. Authors may choose to summarize pairwise comparisons against a chosen treatment included in their analyses for brevity.*  **Discussion/Conclusions:** limitations; conclusions and implications of findings.  **Other:** primary source of funding; systematic review registration number with registry name. | **Pages 2-3** |
|  |  |  |  |
| **INTRODUCTION** |  |  |  |
| Rationale | 3 | Describe the rationale for the review in the context of what is already known*, including mention of why a network meta-analysis has been conducted.* | **Pages 4-5** |
| Objectives | 4 | Provide an explicit statement of questions being addressed, with reference to participants, interventions, comparisons, outcomes, and study design (PICOS). | **Page 5** |
|  |  |  |  |
| **METHODS** |  |  |  |
| Protocol and registration | 5 | Indicate whether a review protocol exists and if and where it can be accessed (e.g., Web address); and, if available, provide registration information, including registration number. | Registered in PROSPERO (CRD420261503119). |
| Eligibility criteria | 6 | Specify study characteristics (e.g., PICOS, length of follow-up) and report characteristics (e.g., years considered, language, publication status) used as criteria for eligibility, giving rationale. *Clearly describe eligible treatments included in the treatment network, and note whether any have been clustered or merged into the same node (with justification).* | Pages 5-6; Table S2 |
| Information sources | 7 | Describe all information sources (e.g., databases with dates of coverage, contact with study authors to identify additional studies) in the search and date last searched. | Pages 5-6 |
| Search | 8 | Present full electronic search strategy for at least one database, including any limits used, such that it could be repeated. | Supplementary Methods |
| Study selection | 9 | State the process for selecting studies (i.e., screening, eligibility, included in systematic review, and, if applicable, included in the meta-analysis). | Page 6; Figure S1 |
| Data collection process | 10 | Describe method of data extraction from reports (e.g., piloted forms, independently, in duplicate) and any processes for obtaining and confirming data from investigators. | Page 6 |
| Data items | 11 | List and define all variables for which data were sought (e.g., PICOS, funding sources) and any assumptions and simplifications made. | Page 6 |
| **Geometry of the network** | **S1** | Describe methods used to explore the geometry of the treatment network under study and potential biases related to it. This should include how the evidence base has been graphically summarized for presentation, and what characteristics were compiled and used to describe the evidence base to readers. | Page 7; Figure 1 |
| Risk of bias within individual studies | 12 | Describe methods used for assessing risk of bias of individual studies (including specification of whether this was done at the study or outcome level), and how this information is to be used in any data synthesis. | Page 6 |
| Summary measures | 13 | State the principal summary measures (e.g., risk ratio, difference in means). *Also describe the use of additional summary measures assessed, such as treatment rankings and surface under the cumulative ranking curve (SUCRA) values, as well as modified approaches used to present summary findings from meta-analyses.* | Pages 6-7 |
| Planned methods of analysis | 14 | Describe the methods of handling data and combining results of studies for each network meta-analysis. This should include, but not be limited to:   - Handling of multi-arm trials; - Selection of variance structure; - Selection of prior distributions in Bayesian analyses; and - Assessment of model fit. | Pages 6-7 |
| **Assessment of Inconsistency** | **S2** | Describe the statistical methods used to evaluate the agreement of direct and indirect evidence in the treatment network(s) studied. Describe efforts taken to address its presence when found. | Page 7 |
| Risk of bias across studies | 15 | Specify any assessment of risk of bias that may affect the cumulative evidence (e.g., publication bias, selective reporting within studies). | Not assessed |
| Additional analyses | 16 | Describe methods of additional analyses if done, indicating which were pre-specified. This may include, but not be limited to, the following:   - Sensitivity or subgroup analyses; - Meta-regression analyses; - Alternative formulations of the treatment network; and - Use of alternative prior distributions for Bayesian analyses (if applicable). | Not assessed |
|  |  |  |  |
| **RESULTS†** |  |  |  |
| Study selection | 17 | Give numbers of studies screened, assessed for eligibility, and included in the review, with reasons for exclusions at each stage, ideally with a flow diagram. | Page 10; Figure S1 |
| **Presentation of network structure** | **S3** | Provide a network graph of the included studies to enable visualization of the geometry of the treatment network. | Figure 1 |
| **Summary of network geometry** | **S4** | Provide a brief overview of characteristics of the treatment network. This may include commentary on the abundance of trials and randomized patients for the different interventions and pairwise comparisons in the network, gaps of evidence in the treatment network, and potential biases reflected by the network structure. | Pages 7 and 10; Figure 1; Table S6 |
| Study characteristics | 18 | For each study, present characteristics for which data were extracted (e.g., study size, PICOS, follow-up period) and provide the citations. | Table S6 |
| Risk of bias within studies | 19 | Present data on risk of bias of each study and, if available, any outcome level assessment. | Figure S16 |
| Results of individual studies | 20 | For all outcomes considered (benefits or harms), present, for each study: 1) simple summary data for each intervention group, and 2) effect estimates and confidence intervals. *Modified approaches may be needed to deal with information from larger networks.* | Table S6; Table 2 |
| Synthesis of results | 21 | Present results of each meta-analysis done, including confidence/credible intervals. *In larger networks, authors may focus on comparisons versus a particular comparator (e.g. placebo or standard care), with full findings presented in an appendix. League tables and forest plots may be considered to summarize pairwise comparisons.* If additional summary measures were explored (such as treatment rankings), these should also be presented. | Page 10; Table 2; Figure 3 |
| **Exploration for inconsistency** | **S5** | Describe results from investigations of inconsistency. This may include such information as measures of model fit to compare consistency and inconsistency models, *P* values from statistical tests, or summary of inconsistency estimates from different parts of the treatment network. | Page 7 |
| Risk of bias across studies | 22 | Present results of any assessment of risk of bias across studies for the evidence base being studied. | Not assessed |
| Results of additional analyses | 23 | Give results of additional analyses, if done (e.g., sensitivity or subgroup analyses, meta-regression analyses*, alternative network geometries studied, alternative choice of prior distributions for Bayesian analyses,* and so forth). | Not assessed |
|  |  |  |  |
| **DISCUSSION** |  |  |  |
| Summary of evidence | 24 | Summarize the main findings, including the strength of evidence for each main outcome; consider their relevance to key groups (e.g., healthcare providers, users, and policy-makers). | Pages 13-14 |
| Limitations | 25 | Discuss limitations at study and outcome level (e.g., risk of bias), and at review level (e.g., incomplete retrieval of identified research, reporting bias). *Comment on the validity of the assumptions, such as transitivity and consistency. Comment on any concerns regarding network geometry (e.g., avoidance of certain comparisons).* | Page 14 |
| Conclusions | 26 | Provide a general interpretation of the results in the context of other evidence, and implications for future research. | Pages 14-15 |
|  |  |  |  |
| **FUNDING** |  |  |  |
| Funding | 27 | Describe sources of funding for the systematic review and other support (e.g., supply of data); role of funders for the systematic review. This should also include information regarding whether funding has been received from manufacturers of treatments in the network and/or whether some of the authors are content experts with professional conflicts of interest that could affect use of treatments in the network. | No funding was received for this study. |

Supplementary Table S2 Inclusion and Exclusion Criteria

| **Literature characteristics** | **Inclusion criteria** | **Exclusion criteria** |
| --- | --- | --- |
| Population | Adult patients with previously untreated advanced  renal cell carcinoma | Patients < 18years old |
|  |  | Healthy subjects |
| Intervention |  | Interventions not for first-line treatment |
|  | Pembrolizumab + Lenvatinib |  |
|  | Nivolumab + Cabozantinib |  |
|  | Avelumab + Axitinib |  |
|  | Benmelstobart + Anlotinib |  |
|  | Atezolizumab + Bevacizumab |  |
|  | Pembrolizumab + Axitinib |  |
|  | Toripalimab + Axitinib |  |
| Comparators | Sunitinib monotherapy | Non-standard regimens |
| Outcomes | Progression-free survival | Patient-reported outcomes |
|  | Overall survival | Biomarker outcomes |
| Study design | Phase Ⅲ Randomized controlled trial (RCT) | Non-RCT |
| Language | English | Non-English |

Supplementary Table S3 CHEERS Checklist.

| **Section/topic** | **Item no.** | **Guidance for reporting** | **Reported in section** |
| --- | --- | --- | --- |
| Title | 1 | Identify the study as an economic evaluation and specify the interventions being compared. | Page 1 |
| Abstract | | | |
| Abstract | 2 | Provide a structured summary that highlights context, key methods, results, and alternative analyses. | Pages 2-3 |
| Introduction | | | |
| Background and objectives | 3 | Give the context for the study, the study question, and its practical relevance for decision making in policy or practice. | Pages 4-5 |
| Methods | | | |
| Health economic analysis plan | 4 | Indicate whether a health economic analysis plan was developed and where available. | Not reported |
| Study population | 5 | Describe characteristics of the study population (such as age range, demographics, socioeconomic, or clinical characteristics). | Pages 5-6; Table S2 |
| Setting and location | 6 | Provide relevant contextual information that may influence findings. | Pages 5 and 8-9 |
| Comparators | 7 | Describe the interventions or strategies being compared and why chosen. | Pages 5-8; Tables 2-3 |
| Perspective | 8 | State the perspective(s) adopted by the study and why chosen. | Pages 2 and 8 |
| Time horizon | 9 | State the time horizon for the study and why appropriate. | Page 7 |
| Discount rate | 10 | Report the discount rate(s) and reason chosen. | Pages 7 and 9 |
| Selection of outcomes | 11 | Describe what outcomes were used as the measure(s) of benefit(s) and harm(s). | Page 9 |
| Measurement of outcomes | 12 | Describe how outcomes used to capture benefit(s) and harm(s) were measured. | Pages 8-9 |
| Valuation of outcomes | 13 | Describe the population and methods used to measure and value outcomes. | Page 8; Table 1 |
| Measurement and valuation of resources and costs | 14 | Describe how costs were valued. | Page 8; Table 1 |
| Currency, price date, and  conversion | 15 | Report the dates of the estimated resource quantities and unit costs, plus the currency and year of conversion. | Pages 8-9; Table 1 |
| Rationale and description of model | 16 | If modeling is used, describe in detail and why used. Report if the model is publicly available and where it can be accessed. | Page 7; Figure 2 |
| Analytics and assumptions | 17 | Describe any methods for analysing or statistically transforming data, any extrapolation methods, and approaches for validating any model used. | Pages 8-10; Tables S4-S5; Figures S2-S15 |
| Characterizing heterogeneity | 18 | Describe any methods used for estimating how the results of the study vary for subgroups. | Not assessed |
| Characterizing distributional effects | 19 | Describe how impacts are distributed across different individuals or adjustments made to reflect priority populations. | Not assessed |
| Characterizing uncertainty | 20 | Describe methods to characterize any sources of uncertainty in the analysis. | Pages 9-10 |
| Approach to engagement with patients and others affected by the study | 21 | Describe any approaches to engage patients or service recipients, the general public, communities, or stakeholders (such as clinicians or payers) in the design of the study. | Not assessed |
| Results | | | |
| Study parameters | 22 | Report all analytic inputs (such as values, ranges, references) including uncertainty or distributional assumptions. | Table 1; Tables S4-S5 |
| Summary of main results | 23 | Report the mean values for the main categories of costs and outcomes of interest and summarize them in the most appropriate overall measure. | Page 11; Table 3 |
| Effect of uncertainty | 24 | Describe how uncertainty about analytic judgments, inputs, or  projections affect findings. Report the effect of choice of  discount rate and time horizon, if applicable. | Pages 11-12; Figures 4-5; Tables 4-6; Table S7 |
| Effect of engagement with patients and others affected by the study | 25 | Report on any difference patient/service recipient, general public, community, or stakeholder involvement made to the approach or findings of the study. | Not assessed |
| Discussion | | | |
| Study findings, limitations, generalizability, and current knowledge | 26 | Report key findings, limitations, ethical or equity considerations not captured, and how these could affect patients, policy, or practice. | Pages 13-15 |
| Other relevant information | | | |
| Source of funding | 27 | Describe how the study was funded and any role of the funder in the identification, design, conduct, and reporting of the analysis | No funding was received for this study. |
| Conflicts of interest | 28 | Report authors conflicts of interest according to journal or International Committee of Medical Journal Editors requirements. | The authors declare no conflicts of interest. |

**Supplementary Table S4. AIC and BIC values for candidate survival models across baseline survival sources**

| **Baseline survival source** | **Distribution** | **PFS AIC** | **PFS BIC** | **OS AIC** | **OS BIC** |
| --- | --- | --- | --- | --- | --- |
| CheckMate 9ER (base case) | Exponential | 1802.32 | 1806.11 | 2145.80 | 2149.59 |
| CheckMate 9ER (base case) | Weibull | 1803.05 | 1810.64 | 2143.71 | 2151.30 |
| CheckMate 9ER (base case) | Gamma | 1798.76 | 1806.34 | 2144.95 | 2152.54 |
| CheckMate 9ER (base case) | Gompertz | 1800.49 | 1808.07 | 2139.90 | 2147.49 |
| CheckMate 9ER (base case) | Lognormal | 1766.14 | 1773.72 | **2138.87** | 2146.46 |
| CheckMate 9ER (base case) | Log-logistic | 1765.74 | 1773.33 | 2139.19 | 2146.77 |
| CheckMate 9ER (base case) | Generalised gamma | 1768.14 | 1779.51 | 2139.46 | 2150.84 |
| CheckMate 9ER (base case) | RP hazard 1 knot | 1762.79 | 1774.17 | 2140.46 | 2151.84 |
| CheckMate 9ER (base case) | RP hazard 2 knots | 1764.81 | 1779.98 | 2141.92 | 2157.09 |
| CheckMate 9ER (base case) | RP hazard 3 knots | 1762.89 | 1781.85 | 2142.63 | 2161.60 |
| CheckMate 9ER (base case) | RP odds 1 knot | 1764.25 | 1775.63 | 2141.13 | 2152.51 |
| CheckMate 9ER (base case) | RP odds 2 knots | 1766.01 | 1781.18 | 2141.30 | 2156.47 |
| CheckMate 9ER (base case) | RP odds 3 knots | 1762.26 | 1781.23 | 2142.59 | 2161.55 |
| CheckMate 9ER (base case) | RP normal 1 knot | 1768.01 | 1779.39 | 2139.12 | 2150.50 |
| CheckMate 9ER (base case) | RP normal 2 knots | 1766.78 | 1781.95 | 2140.45 | 2155.62 |
| CheckMate 9ER (base case) | RP normal 3 knots | **1760.74** | 1779.71 | 2142.00 | 2160.96 |
| CLEAR | Exponential | 1641.53 | 1645.41 | 1541.14 | 1545.01 |
| CLEAR | Weibull | 1643.34 | 1651.09 | 1541.87 | 1549.63 |
| CLEAR | Gamma | 1642.74 | 1650.49 | 1540.75 | 1548.51 |
| CLEAR | Gompertz | 1627.78 | 1635.53 | 1542.67 | 1550.43 |
| CLEAR | Lognormal | 1588.19 | 1595.95 | 1524.73 | 1532.49 |
| CLEAR | Log-logistic | 1599.76 | 1607.51 | 1535.08 | 1542.84 |
| CLEAR | Generalised gamma | 1564.13 | 1575.76 | 1515.46 | 1527.09 |
| CLEAR | RP hazard 1 knot | 1582.71 | 1594.35 | 1523.69 | 1535.32 |
| CLEAR | RP hazard 2 knots | 1580.16 | 1595.67 | 1516.17 | 1531.68 |
| CLEAR | RP hazard 3 knots | 1572.52 | 1591.91 | 1518.33 | 1537.72 |
| CLEAR | RP odds 1 knot | 1581.09 | 1592.73 | 1523.44 | 1535.07 |
| CLEAR | RP odds 2 knots | 1579.43 | 1594.94 | 1515.90 | 1531.42 |
| CLEAR | RP odds 3 knots | 1570.37 | 1589.76 | 1518.12 | 1537.51 |
| CLEAR | RP normal 1 knot | 1571.70 | 1583.34 | 1518.70 | 1530.34 |
| CLEAR | RP normal 2 knots | 1572.10 | 1587.61 | **1513.81** | 1529.32 |
| CLEAR | RP normal 3 knots | **1562.73** | 1582.11 | 1515.97 | 1535.36 |
| ETER100 | Exponential | 1036.07 | 1039.64 | 810.78 | 814.36 |
| ETER100 | Weibull | 1037.09 | 1044.23 | 810.24 | 817.38 |
| ETER100 | Gamma | 1035.54 | 1042.68 | 809.53 | 816.67 |
| ETER100 | Gompertz | 1037.18 | 1044.32 | 812.61 | 819.76 |
| ETER100 | Lognormal | 1018.83 | 1025.97 | **805.45** | 812.60 |
| ETER100 | Log-logistic | 1025.46 | 1032.61 | 807.90 | 815.04 |
| ETER100 | Generalised gamma | 1018.88 | 1029.59 | 807.39 | 818.11 |
| ETER100 | RP hazard 1 knot | 1021.16 | 1031.88 | 806.63 | 817.34 |
| ETER100 | RP hazard 2 knots | 1017.91 | 1032.20 | 808.69 | 822.98 |
| ETER100 | RP hazard 3 knots | 1018.08 | 1035.94 | 807.50 | 825.36 |
| ETER100 | RP odds 1 knot | 1021.82 | 1032.54 | 806.74 | 817.46 |
| ETER100 | RP odds 2 knots | **1016.87** | 1031.16 | 808.77 | 823.06 |
| ETER100 | RP odds 3 knots | 1018.28 | 1036.14 | 807.24 | 825.11 |
| ETER100 | RP normal 1 knot | 1018.72 | 1029.43 | 807.31 | 818.03 |
| ETER100 | RP normal 2 knots | 1018.25 | 1032.54 | 809.20 | 823.49 |
| ETER100 | RP normal 3 knots | 1020.32 | 1038.18 | 807.02 | 824.88 |
| IMmotion151 | Exponential | 2155.53 | 2159.67 | 2650.22 | 2654.35 |
| IMmotion151 | Weibull | 2144.24 | 2152.51 | 2651.68 | 2659.95 |
| IMmotion151 | Gamma | 2136.28 | 2144.55 | 2652.00 | 2660.27 |
| IMmotion151 | Gompertz | 2157.38 | 2165.64 | 2649.95 | 2658.21 |
| IMmotion151 | Lognormal | 2099.30 | 2107.57 | 2650.87 | 2659.14 |
| IMmotion151 | Log-logistic | 2114.32 | 2122.59 | **2648.50** | 2656.77 |
| IMmotion151 | Generalised gamma | 2089.27 | 2101.67 | 2648.95 | 2661.35 |
| IMmotion151 | RP hazard 1 knot | 2094.03 | 2106.43 | 2649.11 | 2661.51 |
| IMmotion151 | RP hazard 2 knots | 2086.08 | 2102.62 | 2650.82 | 2667.35 |
| IMmotion151 | RP hazard 3 knots | **2079.90** | 2100.56 | 2651.29 | 2671.96 |
| IMmotion151 | RP odds 1 knot | 2093.26 | 2105.66 | 2650.47 | 2662.87 |
| IMmotion151 | RP odds 2 knots | 2083.92 | 2100.46 | 2651.28 | 2667.81 |
| IMmotion151 | RP odds 3 knots | 2081.08 | 2101.75 | 2651.69 | 2672.36 |
| IMmotion151 | RP normal 1 knot | 2088.65 | 2101.05 | 2648.99 | 2661.39 |
| IMmotion151 | RP normal 2 knots | 2087.35 | 2103.88 | 2650.74 | 2667.27 |
| IMmotion151 | RP normal 3 knots | 2089.78 | 2110.45 | 2652.04 | 2672.71 |
| JAVELIN Renal 101 | Exponential | 2663.87 | 2667.96 | 2958.13 | 2962.22 |
| JAVELIN Renal 101 | Weibull | 2664.73 | 2672.92 | 2959.32 | 2967.50 |
| JAVELIN Renal 101 | Gamma | 2665.64 | 2673.83 | 2958.44 | 2966.62 |
| JAVELIN Renal 101 | Gompertz | 2645.10 | 2653.29 | 2959.95 | 2968.14 |
| JAVELIN Renal 101 | Lognormal | **2605.82** | 2614.02 | 2958.22 | 2966.41 |
| JAVELIN Renal 101 | Log-logistic | 2612.35 | 2620.54 | **2952.77** | 2960.95 |
| JAVELIN Renal 101 | Generalised gamma | 2606.43 | 2618.71 | 2953.98 | 2966.26 |
| JAVELIN Renal 101 | RP hazard 1 knot | 2607.88 | 2620.17 | 2953.09 | 2965.36 |
| JAVELIN Renal 101 | RP hazard 2 knots | 2608.44 | 2624.82 | 2955.20 | 2971.56 |
| JAVELIN Renal 101 | RP hazard 3 knots | 2607.82 | 2628.29 | 2956.03 | 2976.48 |
| JAVELIN Renal 101 | RP odds 1 knot | 2610.11 | 2622.40 | 2954.68 | 2966.96 |
| JAVELIN Renal 101 | RP odds 2 knots | 2608.83 | 2625.21 | 2956.15 | 2972.51 |
| JAVELIN Renal 101 | RP odds 3 knots | 2608.11 | 2628.59 | 2956.51 | 2976.97 |
| JAVELIN Renal 101 | RP normal 1 knot | 2606.19 | 2618.47 | 2954.63 | 2966.90 |
| JAVELIN Renal 101 | RP normal 2 knots | 2608.10 | 2624.48 | 2956.41 | 2972.78 |
| JAVELIN Renal 101 | RP normal 3 knots | 2609.42 | 2629.90 | 2956.39 | 2976.85 |
| KEYNOTE-426 | Exponential | 2375.67 | 2379.73 | 2836.53 | 2840.59 |
| KEYNOTE-426 | Weibull | 2376.80 | 2384.92 | 2838.30 | 2846.42 |
| KEYNOTE-426 | Gamma | 2377.61 | 2385.73 | 2838.50 | 2846.62 |
| KEYNOTE-426 | Gompertz | 2363.87 | 2371.99 | 2836.79 | 2844.91 |
| KEYNOTE-426 | Lognormal | 2330.40 | 2338.52 | 2833.46 | 2841.59 |
| KEYNOTE-426 | Log-logistic | 2342.73 | 2350.85 | 2834.86 | 2842.98 |
| KEYNOTE-426 | Generalised gamma | 2329.54 | 2341.73 | 2833.51 | 2845.69 |
| KEYNOTE-426 | RP hazard 1 knot | 2334.96 | 2347.14 | 2834.05 | 2846.24 |
| KEYNOTE-426 | RP hazard 2 knots | 2325.81 | 2342.06 | 2833.04 | 2849.29 |
| KEYNOTE-426 | RP hazard 3 knots | 2323.26 | 2343.57 | 2833.72 | 2854.03 |
| KEYNOTE-426 | RP odds 1 knot | 2337.94 | 2350.12 | 2836.66 | 2848.84 |
| KEYNOTE-426 | RP odds 2 knots | **2322.47** | 2338.72 | 2832.56 | 2848.81 |
| KEYNOTE-426 | RP odds 3 knots | 2322.99 | 2343.30 | 2833.85 | 2854.16 |
| KEYNOTE-426 | RP normal 1 knot | 2329.23 | 2341.41 | 2833.68 | 2845.86 |
| KEYNOTE-426 | RP normal 2 knots | 2325.77 | 2342.01 | **2832.30** | 2848.54 |
| KEYNOTE-426 | RP normal 3 knots | 2328.72 | 2349.02 | 2834.22 | 2854.53 |
| RENOTORCH | Exponential | 834.66 | 838.01 | 558.68 | 562.03 |
| RENOTORCH | Weibull | 835.67 | 842.37 | 553.10 | 559.80 |
| RENOTORCH | Gamma | 834.17 | 840.88 | **553.08** | 559.78 |
| RENOTORCH | Gompertz | 835.37 | 842.07 | 554.78 | 561.48 |
| RENOTORCH | Lognormal | 818.67 | 825.37 | 555.88 | 562.58 |
| RENOTORCH | Log-logistic | 824.80 | 831.51 | 553.40 | 560.10 |
| RENOTORCH | Generalised gamma | 816.15 | 826.21 | 555.07 | 565.12 |
| RENOTORCH | RP hazard 1 knot | 817.88 | 827.94 | 554.96 | 565.01 |
| RENOTORCH | RP hazard 2 knots | 818.08 | 831.49 | 556.88 | 570.28 |
| RENOTORCH | RP hazard 3 knots | 817.02 | 833.78 | 556.19 | 572.95 |
| RENOTORCH | RP odds 1 knot | 817.67 | 827.73 | 555.40 | 565.45 |
| RENOTORCH | RP odds 2 knots | 817.19 | 830.60 | 557.20 | 570.61 |
| RENOTORCH | RP odds 3 knots | 817.00 | 833.76 | 556.31 | 573.07 |
| RENOTORCH | RP normal 1 knot | **816.11** | 826.17 | 555.50 | 565.56 |
| RENOTORCH | RP normal 2 knots | 817.38 | 830.79 | 557.40 | 570.81 |
| RENOTORCH | RP normal 3 knots | 818.47 | 835.23 | 556.30 | 573.06 |

AIC, Akaike information criterion; BIC, Bayesian information criterion; OS, overall survival; PFS, progression-free survival; RP, Royston–Parmar.

**Supplementary Table S5. Selected survival distributions and parameter estimates for baseline survival extrapolation**

| **Baseline survival source** | **Endpoint** | **Selected distribution** | **Parameter estimates** |
| --- | --- | --- | --- |
| CheckMate 9ER (base case) | OS | Lognormal | meanlog = 3.527439; sdlog = 1.553262 |
| CheckMate 9ER (base case) | PFS | RP normal 3 knots | gamma0 = -2.1476581; gamma1 = 0.5228096; gamma2 = -0.4391921; gamma3 = 0.7159172; gamma4 = -0.3570750 |
| CLEAR | OS | RP normal 2 knots | gamma0 = -4.2258905; gamma1 = 1.4316206; gamma2 = 0.4031320; gamma3 = -0.3660807 |
| CLEAR | PFS | RP normal 3 knots | gamma0 = -2.7535; gamma1 = 2.0075; gamma2 = 0.9792; gamma3 = -0.7320; gamma4 = 0.2637 |
| ETER100 | OS | Lognormal | meanlog = 3.781016; sdlog = 1.382600 |
| ETER100 | PFS | RP odds 2 knots | gamma0 = -4.0107; gamma1 = 3.4678; gamma2 = 0.4395; gamma3 = -0.3937 |
| IMmotion151 | OS | Log-logistic | shape = 1.176172; scale = 31.121254 |
| IMmotion151 | PFS | RP hazard 3 knots | gamma0 = -5.9978394; gamma1 = 5.7365279; gamma2 = 1.7797538; gamma3 = -1.8778072; gamma4 = 0.8450706 |
| JAVELIN Renal 101 | OS | Log-logistic | shape = 1.344721; scale = 37.629934 |
| JAVELIN Renal 101 | PFS | Lognormal | meanlog = 2.1997; sdlog = 1.1350 |
| KEYNOTE-426 | OS | RP normal 2 knots | gamma0 = -2.4899542; gamma1 = 0.7300384; gamma2 = 0.0776467; gamma3 = -0.1529248 |
| KEYNOTE-426 | PFS | RP odds 2 knots | gamma0 = -4.2343205; gamma1 = 3.1163679; gamma2 = 0.3811887; gamma3 = -0.3703746 |
| RENOTORCH | OS | Gamma | shape = 1.54536780; rate = 0.04115951 |
| RENOTORCH | PFS | RP normal 1 knot | gamma0 = -2.17204817; gamma1 = 1.19661348; gamma2 = 0.04939744 |

Abbreviations: OS, overall survival; PFS, progression-free survival; RP, Royston-Parmar.

Supplementary Table S6 Characteristics of the randomized controlled trials included in the network meta-analysis.

| **Study** | **Author** | **Year** | **Study design** | **Sample size** | **Patient** | **Intervention** | **Control** | **HR of PFS (95% CI)** | **HR of OS (95% CI)** |
| --- | --- | --- | --- | --- | --- | --- | --- | --- | --- |
| CLEAR | Robert J. Motzer. et al | 2024 | RCT | 355/357 | RCC | Pembrolizumab + Lenvatinib | Sunitinib | 0.47 (0.38-0.57) | 0.79 (0.63-0.99) |
| Checkmate 9ER | R. J. Motzer. et al | 2025 | RCT | 323/328 | RCC | Nivolumab + Cabozantinib | Sunitinib | 0.58 (0.49-0.70) | 0.79 (0.65-0.96) |
| JAVELIN Renal 101 | T. K. Choueiri. et al | 2024 | RCT | 442/444 | RCC | Avelumab + Axitinib | Sunitinib | 0.66 (0.566-0.769) | 0.88 (0.749-1.039） |
| ETER100 | [Aiping Zhou. et al](https://pubmed.ncbi.nlm.nih.gov/?size=200&term=Zhou+A&cauthor_id=40907513) | 2025 | RCT | 264/263 | RCC | Benmelstobart + Anlotinib | Sunitinib | 0.53 (0.42-0.67) | 0.66（0.48-0.92） |
| IMmotion151 | R.J. Motzer. et al | 2022 | RCT | 454/461 | RCC | Atezolizumab + Bevacizumab | Sunitinib | 0.83（0.70-0.97） | 0.91 (0.76-1.08) |
| KEYNOTE-426 | [Brian I Rini](https://pubmed.ncbi.nlm.nih.gov/?term=Rini+BI&cauthor_id=40750932). et al | 2025 | RCT | 432/429 | RCC | Pembrolizumab + Axitinib | Sunitinib | 0.69 (0.59-0.81) | 0.84 (0.71-0.99) |
| RENOTORCH | X Q Yan.et al | 2023 | RCT | 210/211 | RCC | Toripalimab + Axitinib | Sunitinib | 0.65(0.49-0.86） | 0.61（0.40-0.92） |

**Supplementary Table S7. Cost-effectiveness results under alternative baseline survival assumptions**

| **Baseline survival source** | **Treatment** | **Total cost (US$)** | **Incremental cost (US$)** | **QALYs** | **Incremental QALYs** | **ICER (US$/QALY)** |
| --- | --- | --- | --- | --- | --- | --- |
| **CLEAR** | Sunitinib | 97,525.07 | — | 4.0522 | — | — |
| CLEAR | Toripalimab + Axitinib | 234,170.21 | 136,645.13 | 5.8209 | 1.7688 | 77,254.38 |
| CLEAR | Atezolizumab + Bevacizumab | 379,171.28 | 281,646.20 | 4.4272 | 0.3750 | 751,078.78 |
| CLEAR | Benmelstobart + Anlotinib | 426,763.60 | 329,238.52 | 5.6910 | 1.6388 | 200,901.56 |
| CLEAR | Pembrolizumab + Axitinib | 513,060.36 | 415,535.29 | 4.7734 | 0.7212 | 576,145.23 |
| CLEAR | Pembrolizumab + Lenvatinib | 523,097.23 | 425,572.15 | 5.1912 | 1.1391 | 373,617.52 |
| CLEAR | Avelumab + Axitinib | 700,789.46 | 603,264.39 | 4.6498 | 0.5977 | 1,009,366.62 |
| CLEAR | Nivolumab + Cabozantinib | 1,538,732.93 | 1,441,207.86 | 5.0658 | 1.0136 | 1,421,812.45 |
| **ETER100** | Sunitinib | 115,496.06 | — | 4.4613 | — | — |
| ETER100 | Toripalimab + Axitinib | 239,941.55 | 124,445.49 | 6.1416 | 1.6803 | 74,060.57 |
| ETER100 | Atezolizumab + Bevacizumab | 376,392.20 | 260,896.14 | 4.8162 | 0.3550 | 735,007.36 |
| ETER100 | Benmelstobart + Anlotinib | 419,133.03 | 303,636.96 | 6.0121 | 1.5508 | 195,789.37 |
| ETER100 | Pembrolizumab + Axitinib | 500,621.40 | 385,125.33 | 5.1419 | 0.6806 | 565,835.27 |
| ETER100 | Pembrolizumab + Lenvatinib | 506,196.24 | 390,700.18 | 5.5320 | 1.0707 | 364,894.93 |
| ETER100 | Avelumab + Axitinib | 668,695.22 | 553,199.16 | 5.0205 | 0.5592 | 989,244.84 |
| ETER100 | Nivolumab + Cabozantinib | 1,341,318.37 | 1,225,822.31 | 5.4165 | 0.9552 | 1,283,307.22 |
| **IMmotion151** | Sunitinib | 95,961.50 | — | 3.6969 | — | — |
| IMmotion151 | Toripalimab + Axitinib | 205,951.96 | 109,990.47 | 5.3646 | 1.6677 | 65,952.01 |
| IMmotion151 | Atezolizumab + Bevacizumab | 308,902.81 | 212,941.32 | 4.0309 | 0.3340 | 637,515.30 |
| IMmotion151 | Benmelstobart + Anlotinib | 346,789.91 | 250,828.42 | 5.1985 | 1.5017 | 167,034.56 |
| IMmotion151 | Pembrolizumab + Axitinib | 407,381.96 | 311,420.46 | 4.3371 | 0.6402 | 486,422.11 |
| IMmotion151 | Pembrolizumab + Lenvatinib | 411,851.21 | 315,889.72 | 4.6861 | 0.9893 | 319,319.39 |
| IMmotion151 | Avelumab + Axitinib | 539,285.56 | 443,324.06 | 4.2096 | 0.5127 | 864,691.31 |
| IMmotion151 | Nivolumab + Cabozantinib | 951,967.41 | 856,005.91 | 4.5943 | 0.8974 | 953,876.38 |
| **JAVELIN Renal 101** | Sunitinib | 106,477.11 | — | 3.9508 | — | — |
| JAVELIN Renal 101 | Toripalimab + Axitinib | 212,637.01 | 106,159.90 | 5.5693 | 1.6184 | 65,594.65 |
| JAVELIN Renal 101 | Atezolizumab + Bevacizumab | 315,333.59 | 208,856.48 | 4.2773 | 0.3265 | 639,709.26 |
| JAVELIN Renal 101 | Benmelstobart + Anlotinib | 357,606.32 | 251,129.21 | 5.4202 | 1.4693 | 170,914.98 |
| JAVELIN Renal 101 | Pembrolizumab + Axitinib | 416,864.25 | 310,387.13 | 4.5788 | 0.6279 | 494,301.47 |
| JAVELIN Renal 101 | Pembrolizumab + Lenvatinib | 427,801.27 | 321,324.15 | 4.9345 | 0.9836 | 326,672.85 |
| JAVELIN Renal 101 | Avelumab + Axitinib | 550,398.85 | 443,921.74 | 4.4578 | 0.5069 | 875,688.91 |
| JAVELIN Renal 101 | Nivolumab + Cabozantinib | 1,013,848.21 | 907,371.10 | 4.8344 | 0.8835 | 1,026,993.14 |
| **KEYNOTE-426** | Sunitinib | 99,455.05 | NA | 4.0480 | NA | NA |
| KEYNOTE-426 | Toripalimab + Axitinib | 225,545.70 | 126,090.64 | 5.6852 | 1.6372 | 77,014.56 |
| KEYNOTE-426 | Atezolizumab + Bevacizumab | 362,871.25 | 263,416.20 | 4.3867 | 0.3387 | 777,748.19 |
| KEYNOTE-426 | Benmelstobart + Anlotinib | 399,505.00 | 300,049.94 | 5.5535 | 1.5055 | 199,303.04 |
| KEYNOTE-426 | Pembrolizumab + Axitinib | 481,476.31 | 382,021.25 | 4.7006 | 0.6526 | 585,341.48 |
| KEYNOTE-426 | Pembrolizumab + Lenvatinib | 488,469.07 | 389,014.01 | 5.0810 | 1.0330 | 376,590.04 |
| KEYNOTE-426 | Avelumab + Axitinib | 653,096.22 | 553,641.16 | 4.5843 | 0.5364 | 1,032,211.01 |
| KEYNOTE-426 | Nivolumab + Cabozantinib | 1,306,870.03 | 1,207,414.97 | 4.9675 | 0.9195 | 1,313,105.89 |
| **RENOTORCH** | Sunitinib | 52,028.77 | NA | 2.8966 | NA | NA |
| RENOTORCH | Toripalimab + Axitinib | 176,887.08 | 124,858.31 | 4.0459 | 1.1493 | 108,639.95 |
| RENOTORCH | Benmelstobart + Anlotinib | 322,187.97 | 270,159.21 | 3.9156 | 1.0190 | 265,131.45 |
| RENOTORCH | Atezolizumab + Bevacizumab | 328,747.66 | 276,718.89 | 3.1212 | 0.2245 | 1,232,363.31 |
| RENOTORCH | Pembrolizumab + Lenvatinib | 373,356.99 | 321,328.23 | 3.5263 | 0.6297 | 510,291.13 |
| RENOTORCH | Pembrolizumab + Axitinib | 418,170.85 | 366,142.08 | 3.3259 | 0.4293 | 852,949.73 |
| RENOTORCH | Avelumab + Axitinib | 577,885.38 | 525,856.61 | 3.2414 | 0.3448 | 1,525,151.88 |
| RENOTORCH | Nivolumab + Cabozantinib | 1,027,844.65 | 975,815.88 | 3.4922 | 0.5956 | 1,638,469.57 |

ICER, incremental cost-effectiveness ratio; QALY, quality-adjusted life year.

Supplementary Figure S1 Flowchart of study selection.

Supplementary Figure S2 Reconstructed Kaplan–Meier curve and candidate survival extrapolation models for overall survival in the sunitinib arm of CheckMate 9ER

Supplementary Figure S3 Reconstructed Kaplan–Meier curve and candidate survival extrapolation models for Progression‑Free Survival in the sunitinib arm of CheckMate 9ER

Supplementary Figure S4 Reconstructed Kaplan–Meier curve and candidate survival extrapolation models for overall survival in the sunitinib arm of CLEAR

Supplementary Figure S5 Reconstructed Kaplan–Meier curve and candidate survival extrapolation models for Progression‑Free Survival in the sunitinib arm of CLEAR

Supplementary Figure S6 Reconstructed Kaplan–Meier curve and candidate survival extrapolation models for overall survival in the sunitinib arm of JAVELIN Renal 101

Supplementary Figure S7 Reconstructed Kaplan–Meier curve and candidate survival extrapolation models for Progression‑Free Survival in the sunitinib arm of JAVELIN Renal 101

Supplementary Figure S8 Reconstructed Kaplan–Meier curve and candidate survival extrapolation models for overall survival in the sunitinib arm of ETER100

Supplementary Figure S9 Reconstructed Kaplan–Meier curve and candidate survival extrapolation models for Progression‑Free Survival in the sunitinib arm of ETER100

Supplementary Figure S10 Reconstructed Kaplan–Meier curve and candidate survival extrapolation models for overall survival in the sunitinib arm of IMmotion151

Supplementary Figure S11 Reconstructed Kaplan–Meier curve and candidate survival extrapolation models for Progression‑Free Survival in the sunitinib arm of IMmotion151

Supplementary Figure S12 Reconstructed Kaplan–Meier curve and candidate survival extrapolation models for overall survival in the sunitinib arm of KEYNOTE-426

Supplementary Figure S13 Reconstructed Kaplan–Meier curve and candidate survival extrapolation models for Progression‑Free Survival in the sunitinib arm of KEYNOTE-426

Supplementary Figure S14 Reconstructed Kaplan–Meier curve and candidate survival extrapolation models for overall survival in the sunitinib arm of RENOTORCH

Supplementary Figure S15 Reconstructed Kaplan–Meier curve and candidate survival extrapolation models for Progression‑Free Survival in the sunitinib arm of RENOTORCH

Supplementary Figure S16 Risk of Summary

Supplementary Methods. Search strategies for PubMed, EMBASE and Cochrane database of network meta-analysis.

Pubmed

#1 "Carcinoma, Renal Cell"[Mesh] OR "Renal Cell Carcinoma"[tiab] OR RCC[tiab] OR "Clear Cell Renal Cell Carcinoma"[tiab] OR ccRCC[tiab] OR "Papillary Renal Cell Carcinoma"[tiab] OR "Chromophobe Renal Cell Carcinoma"[tiab] OR "Metastatic Renal Cell Carcinoma"[tiab] OR mRCC[tiab] OR (first-line[tiab] AND ("Renal Cell Carcinoma"[tiab] OR RCC[tiab])) OR (frontline[tiab] AND ("Renal Cell Carcinoma"[tiab] OR RCC[tiab])) OR (1st-line[tiab] AND ("Renal Cell Carcinoma"[tiab] OR RCC[tiab]))

#2 "Immunotherapy"[Mesh] OR Immunotherap*[tiab] OR immunolog* therap*[tiab] OR immune checkpoint inhibitor*[tiab] OR ICIs[tiab] OR immune checkpoint blockade*[tiab] OR immunocheckpoint inhibitor*[tiab] OR check-point inhibitor*[tiab] OR CPIs[tiab] OR "Programmed Cell Death 1 Receptor"[Mesh] OR "B7-H1 Antigen"[Mesh] OR programmed cell death protein-1[tiab] OR programmed cell death-ligand 1[tiab] OR "PD-1"[tiab] OR "PD-L1"[tiab] OR "PD1"[tiab] OR "PDL1"[tiab] OR cytotoxic T lymphocyte-associated protein-4 inhibitor*[tiab] OR CTLA-4 inhibitor*[tiab] OR CTLA-4 antibod*[tiab] OR CTLA-4 anti-bod*[tiab] OR "anti-CTLA-4"[tiab] OR CTLA4 inhibitor*[tiab] OR CTLA4 antibod*[tiab] OR CTLA4 anti-bod*[tiab] OR "anti-CTLA4"[tiab] OR "CTLA-4 Antigen"[Mesh] OR CD152 Antigen*[tiab] OR CTLA 4 Antigen*[tiab] OR CTLA4 Antigen*[tiab]

#3 ("Lymphocyte Activation Gene 3 Protein"[Mesh] OR "T-cell Immunoglobulin and Mucin Domain Containing 3 Protein"[Mesh] OR "lymphocyte activation gene 3"[tw] OR Lag3[tw] OR "LAG-3"[tw] OR "TIM-3"[tw] OR TIGIT[tw] OR "T-cell immunoreceptor with Ig and ITIM domains"[tw] OR VISTA[tw] OR "B7-H3"[tw] OR "CD276"[tw]) AND (inhibitor*[tw] OR anti-bod*[tw] OR antibod*[tw] OR blockade*[tw] OR agonist*[tw])

#4 "Nivolumab"[Mesh] OR Nivolumab[tw] OR Opdivo[tw] OR "BMS-936558"[tw] OR "Pembrolizumab"[Supplementary Concept] OR Pembrolizumab[tw] OR Keytruda[tw] OR "MK-3475"[tw] OR "Atezolizumab"[Supplementary Concept] OR Atezolizumab[tw] OR Tecentriq[tw] OR "MPDL3280A"[tw] OR "Durvalumab"[Supplementary Concept] OR Durvalumab[tw] OR Imfinzi[tw] OR "MEDI4736"[tw] OR "Avelumab"[Supplementary Concept] OR Avelumab[tw] OR Bavencio[tw] OR "MSB0010682"[tw] OR "Ipilimumab"[Mesh] OR Ipilimumab[tw] OR Yervoy[tw] OR "MDX-010"[tw] OR "Cemiplimab"[Supplementary Concept] OR Cemiplimab[tw] OR Libtayo[tw] OR "REGN2810"[tw] OR "Relatlimab"[Supplementary Concept] OR Relatlimab[tw] OR "BMS-986016"[tw]

#5 "Angiogenesis Inhibitors"[Mesh] OR "Protein Kinase Inhibitors"[Mesh] OR "mTOR Inhibitors"[Mesh] OR target* therap*[tiab] OR Tyrosine Kinase Inhibitor*[tiab] OR TKI*[tiab] OR anti-angiogen*[tiab] OR VEGF[tiab] OR VEGFR[tiab] OR VEGFR-1[tiab] OR VEGFR-2[tiab] OR VEGFR-3[tiab] OR mTOR[tiab] OR PI3K[tiab] OR AKT[tiab] OR Sunitinib[tw] OR Sutent[tw] OR "SU11248"[tw] OR Pazopanib[tw] OR Votrient[tw] OR "GW786034"[tw] OR Cabozantinib[tw] OR Cabometyx[tw] OR "XL184"[tw] OR Axitinib[tw] OR Inlyta[tw] OR "AG-013736"[tw] OR Lenvatinib[tw] OR Lenvima[tw] OR "E7080"[tw] OR Tivozanib[tw] OR Fotivda[tw] OR Everolimus[tw] OR Afinitor[tw] OR "RAD001"[tw] OR Temsirolimus[tw] OR Torisel[tw] OR "CCI-779"[tw] OR Belzutifan[tw] OR Welireg[tw]

#6 ("controlled clinical trial"[pt] OR "Controlled Clinical Trials as Topic"[MeSH] OR "Random Allocation"[MeSH] OR "Double-Blind Method"[MeSH] OR "single-blind method"[MeSH] OR "Control Groups"[MeSH] OR "cross-over studies"[MeSH] OR random*[tiab] OR placebo[tiab] OR trial[tiab] OR groups[tiab] OR crossover[tiab] OR cross-over[tiab]) NOT ("Animals"[Mesh] NOT ("Humans"[Mesh] AND "Animals"[Mesh]))

#7 #2 OR #3 OR #4 OR #5

#8 #1 AND #7 AND #6

Embase

#1 'renal cell carcinoma'/exp OR ((renal OR kidney) NEAR/4 'renal cell' NEAR/4 (cancer OR carcinoma) AND (first-line OR 'first line' OR frontline OR 1st-line OR metastatic OR advanced-stage OR 'advanced stage' OR mRCC)):ab,ti,kw

#2 'immunotherapy'/exp OR (Immunotherap* OR immunolog* NEAR/3 therap* OR "CAR T-Cell Therap*" OR immune* NEAR/3 (checkpoint OR check-point) NEAR/3 (inhibitor* OR suppressor* OR blockade*) OR immunocheckpoint NEAR/3 (inhibitor* OR suppressor* OR blockade*) OR ICIs OR CPIs):ab,ti,kw

#3 'programmed death 1 receptor'/exp OR 'programmed death 1 ligand 2'/exp OR 'programmed death 1 ligand 1'/exp OR 'Nivolumab'/exp OR 'atezolizumab'/exp OR 'pembrolizumab'/exp OR 'cemiplimab'/exp OR 'avelumab'/exp OR 'durvalumab'/exp OR 'cytotoxic T lymphocyte antigen 4'/exp OR 'Ipilimumab'/exp OR 'ticilimumab'/exp OR 'relatlimab'/exp

#4 (programmed NEAR/1 death NEAR/2 1 OR "PD-1" OR "PD-L1" OR "PD1" OR "PDL1" OR ("cytotoxic T lymphocyte-associated protein-4" OR CTLA-4 OR CTLA4 OR CD152 OR CD223 OR "lymphocyte activation gene 3" OR Lag3 OR "LAG-3" OR "T cell immunoglobulin-3" OR "TIM-3" OR TIGIT) NEAR/3 (Antigen* OR anti OR inhibitor* OR anti-bod* OR antibod*)):ab,ti,kw

#5 (Nivolumab OR Opdivo OR "BMS-936558" OR pembrolizumab OR Keytruda OR "MK-3475" OR atezolizumab OR Tecentriq OR MPDL3280A OR durvalumab OR Imfinzi OR MEDI4736 OR avelumab OR Bavencio OR Ipilimumab OR Yervoy OR relatlimab OR BMS986016 OR Sunitinib OR Sutent OR Pazopanib OR Votrient OR Cabozantinib OR Cabometyx OR Axitinib OR Inlyta OR Lenvatinib OR Lenvima OR Everolimus OR Afinitor):ab,ti,kw

#6 #1 AND (#2 OR #3 OR #4 OR #5)

#7 (('controlled clinical trial'/exp OR 'Controlled Clinical Trial (Topic)'/exp OR 'double blind procedure'/de OR 'control group'/de OR 'crossover procedure'/de OR 'single blind procedure'/de OR 'triple blind procedure'/de OR 'placebo'/de OR 'randomization'/exp) OR (random* OR trial OR groups OR placebo* OR crossover OR "cross-over"):ab,ti,kw) AND 'human'/exp

#8 #6 AND #7

Cochrane

#1. MeSH descriptor: [Kidney Neoplasms] explode all trees

#2. MeSH descriptor: [Carcinoma, Renal Cell] explode all trees

#3. (renal OR kidney) AND (cell AND (carcinoma OR cancer OR adenocarcinoma)) AND (advanced OR metastatic OR mRCC OR "stage IV" OR unresectable)

#4. "advanced renal cell carcinoma" OR "metastatic renal cell carcinoma" OR aRCC OR mRCC OR "clear cell RCC"

#5. #1 OR #2 OR #3 OR #4

#6. MeSH descriptor: [Immune Checkpoint Inhibitors] explode all trees

#7. MeSH descriptor: [Nivolumab] explode all trees

#8. MeSH descriptor: [Pembrolizumab] explode all trees

#9. MeSH descriptor: [Ipilimumab] explode all trees

#10. Immunotherapy OR "immune checkpoint inhibitor*" OR ICI OR CPI OR "anti-PD-1" OR "anti-PD-L1" OR "anti-CTLA-4" OR "programmed death 1" OR "programmed cell death 1 ligand 1"

#11. nivolumab OR pembrolizumab OR ipilimumab OR atezolizumab OR avelumab OR durvalumab OR toripalimab OR sintilimab OR camrelizumab OR tislelizumab

#12. #6 OR #7 OR #8 OR #9 OR #10 OR #11

#13. MeSH descriptor: [Axitinib] explode all trees

#14. MeSH descriptor: [Cabozantinib] explode all trees

#15. MeSH descriptor: [Lenvatinib] explode all trees

#16. MeSH descriptor: [Sunitinib] explode all trees

#17. tyrosine kinase inhibitor OR TKI OR VEGFR inhibitor OR vascular endothelial growth factor receptor inhibitor

#18. axitinib OR cabozantinib OR lenvatinib OR pazopanib OR tivozanib OR sorafenib

#19. #13 OR #14 OR #15 OR #16 OR #17 OR #18

#20. #12 AND #19

#21. randomized controlled trial OR randomised controlled trial

#22. #5 AND #20 AND #2
